# Supplementary material for: Feasibility of Invasive Brown Seaweed Rugulopteryx okamurae as Source of Alginate: Characterization of Products and Evaluation of Derived Gels
Source: Polymers (Basel). 2024 Mar 5;16(5):702. doi: 10.3390/polym16050702 (PMC10934190; doi:10.3390/polym16050702)
Supplement: Supplementary file 1 [file polymers-16-00702-s001.zip › polymers-2841113-supplementary.pdf]

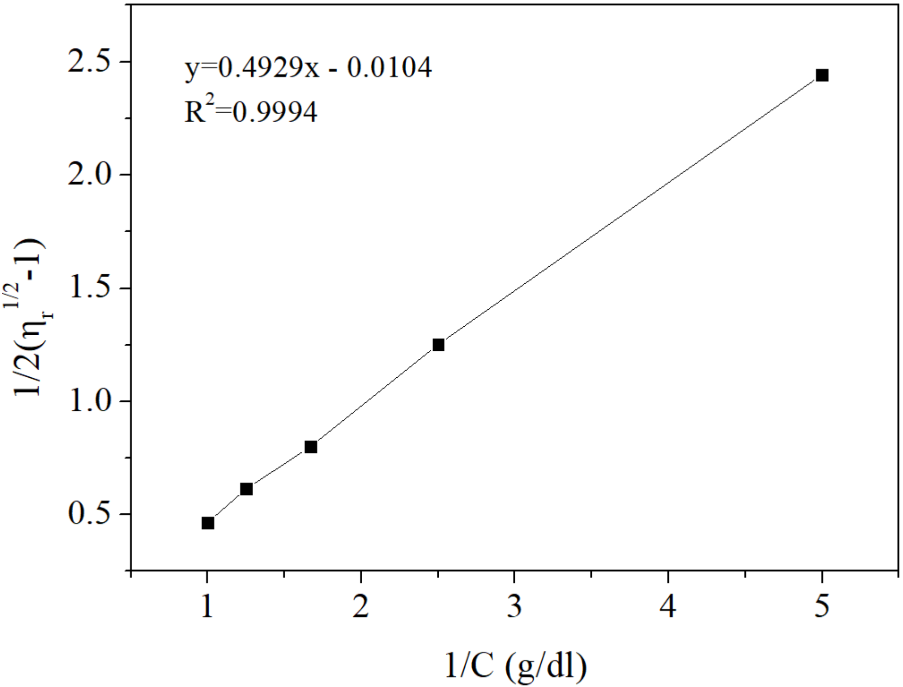

Figure S1. Correlation between viscosity and the inverse of concentration for RO-extracted alginate solutions at different concentrations following Fedors' Equation (1).
